# GravelSens: A Smart Gravel Sensor for High-Resolution, Non-Destructive Monitoring of Clogging Dynamics

**DOI:** 10.3390/s25020536

**Published:** 2025-01-17

**Authors:** Kaan Koca, Eckhard Schleicher, André Bieberle, Stefan Haun, Silke Wieprecht, Markus Noack

**Affiliations:** 1 Institute for Modelling Hydraulic and Environmental Systems, University of Stuttgart, 70569 Stuttgart, Germany; stefan.haun@iws.uni-stuttgart.de (S.H.); silke.wieprecht@iws.uni-stuttgart.de (S.W.); 2 Faculty of Architecture and Civil Engineering, Karlsruhe University of Applied Sciences, 76133 Karlsruhe, Germany; markus.noack@h-ka.de; 3 Helmholtz-Zentrum Dresden—Rossendorf, 01328 Dresden, Germany; e.schleicher@hzdr.de (E.S.); a.bieberle@hzdr.de (A.B.)

**Keywords:** wire-mesh sensor, conductance sensor, pore-filling measurement, two-phase flow, porous media, gravel bed, fine sediment, clogging, sediment deposition, sediment transport

## Abstract

Engineers, geomorphologists, and ecologists acknowledge the need for temporally and spatially resolved measurements of sediment clogging (also known as colmation) in permeable gravel-bed rivers due to its adverse impacts on water and habitat quality. In this paper, we present a novel method for non-destructive, real-time measurements of pore-scale sediment deposition and monitoring of clogging by using wire-mesh sensors (WMSs) embedded in spheres, forming a smart gravel bed (GravelSens). The measuring principle is based on one-by-one voltage excitation of transmitter electrodes, followed by simultaneous measurements of the resulting current by receiver electrodes at each crossing measuring pores. The currents are then linked to the conductive component of fluid impedance. The measurement performance of the developed sensor is validated by applying the Maxwell Garnett and parallel models to sensor data and comparing the results to data obtained by gamma ray computed tomography (CT). GravelSens is tested and validated under varying filling conditions of different particle sizes ranging from sand to fine gravel. The close agreement between GravelSens and CT measurements indicates the technology’s applicability in sediment–water research while also suggesting its potential for other solid–liquid two-phase flows. This pore-scale measurement and visualization system offers the capability to monitor clogging and de-clogging dynamics within pore spaces up to 10,000 Hz, making it the first laboratory equipment capable of performing such in situ measurements without radiation. Thus, GravelSens is a major improvement over existing methods and holds promise for advancing the understanding of flow–sediment–ecology interactions.

## 1. Introduction

Fine sediments are a fundamental component of the gravel–cobble substrate of river systems [1,2], also supporting healthy riverine functioning [3,4,5]. However, fine sediment delivery to river corridors continues to rise globally due to anthropogenic disturbances (e.g., intensive agriculture and mining) and the impact of climate change [6,7,8], resulting in the deposition of excess fine sediment in freshwater habitats. The infiltration and deposition of fine sediment in gravel-bed rivers is known as clogging or colmation [9]. Clogging is a progressive and dynamic process that is characterized by high spatial and temporal variability. Depending on local geomorphological and hydraulic variables, clogging occurs unimpeded by deep infiltration (i.e., unimpeded static percolation) or is restricted to a certain vertical depth due to bridging and accumulation processes [4]. By reducing bed permeability, interstitial flow, and thus connectivity, clogging hampers surface–subsurface (hyporheic) exchange of water, dissolved oxygen, and nutrients [4,10]. As a result, it can have detrimental effects on benthic invertebrates [11,12] and fish populations in gravel-bed rivers [13,14,15], which use interstices (pores) as habitat, as well as on macrophytes [16] which represent the base food chain for these organisms. Due to sediment-bound heavy metals and pollutants, clogging can also have deleterious effects on water quality [17,18]. Therefore, understanding the mechanisms of fine sediment infiltration and clogging of gravel-bed rivers is fundamental to effectively maintaining and managing the ecological health and water quality within these ecosystems.

Despite its importance and the well-documented need for understanding [4,10], the mechanisms inducing clogging and its opposite process of de-clogging in gravel-bed rivers remain poorly understood. This is due to the lack of data on clogging dynamics at the pore scale, where fine sediments deposit. Existing measurement techniques are incapable of providing such data with sufficient temporal and spatial resolution.

While suspended sediments can be continuously quantified with turbidity measurements [19,20], there exists no standardized technique to quantify fine sediment clogging in gravel beds consistently. Various techniques have been used to assess clogging in both the laboratory and in the field. In the field, disturbance/resuspension approaches [21,22], bulk core sampling [23], freeze coring [24,25,26,27,28], and sediment traps [21,29,30,31,32,33] are commonly employed, mostly in an event-based manner (e.g., before and after flood). Similarly, cylindrical core samplers [34] and different versions of in situ sediment traps, including solid-walled [35,36], semi-permeable [37], and gravel-filled [38] containers, are utilized in laboratory flume studies. Yet, these techniques only allow for bulk sampling and provide only spatially averaged and time-integrated (ranging from a few weeks to months in the field, or from a few hours to days in laboratories) snapshots of the clogging situation [19,39]. Additionally, the sampling strategy is often labor-intensive and destructive to the sample or the surrounding environment. Each technique has also distinct limitations in terms of environmental representativeness, comparability, bias (e.g., exclusion of lateral sediment transport), and repeatability (see reviews [40,41,42,43,44] for more detail).

In an attempt to overcome these issues, non-destructive techniques have also been applied. These techniques include ionizing radiation-based X-ray micro-computed tomography (micro-CT) [45] and gamma ray attenuation (GRA) [46], as well as electromagnetic radiation-based magnetic resonance imaging (MRI) [47]. These techniques, however, have prohibitively high costs and usually require sophisticated setup within special containment facilities, preventing their in situ deployment at hydraulic flumes. While micro-CT and MRI techniques provide three-dimensional measurements of clogging at high and low spatial resolutions, respectively, they require retraction of sediment samples from the flume and their transfer to the measurement facility [45,47], preventing their application for monitoring real-time dynamic clogging processes and making the sampling disruptive. Even though GRA can be used in open laboratory environments at relatively low source activities [46,48], its deployment in flumes is also limited to certain substrate density and sediment deposition conditions due to the radiation-related limitations and safety issues. Furthermore, GRA only provides integrated measurements across a cross-section of the flume, lacking the capability to capture a spatio-temporal distribution of fine sediment clogging, which is important for understanding and assessing its dynamics. Ultimately, there is a need for rapid, robust, flexible, and cost-effective methods capable of monitoring the spatial and temporal evolution of clogging in the gravel bed as a response to changing environmental conditions [4,10,19,21].

A promising technique is the so-called wire-mesh sensor (WMS) [49,50] technique that is based on measurements of electrical signals in virtual wire crossings associated with local electrical impedance distribution. Such sensors have mostly been utilized to measure void fractions and velocities in multiphase flows, with various applications in the fields of chemical and petroleum industries (see reviews [51,52]). Given that the WMS technique can quantify conductance, it holds promise for potentially relating conductance fluctuations to sediment deposition in pore spaces, which can also be referred to as “degree of clogging”. In a previous study, a capacitance-based WMS was utilized to measure the sand concentration in a stirred reactor [53]. This approach requires demineralized water, which is difficult to achieve in flume studies due to continuous sediment supply. Additionally, such an approach is intrusive due to the direct placement of wires within the gravel bed, which would considerably block the interstitial space, preventing sediment infiltration and additionally altering the local hydraulics. Electrical impedance or resistance can also be measured using similar popular techniques, such as electrical resistivity or capacitance tomography. However, since these techniques require placing electrodes on the walls of the flume, the measurement accuracy and spatio-temporal resolution are strongly altered by non-linear properties of the electromagnetic field [54,55].

In this study, we present a novel, non-destructive, smart gravel sensor (GravelSens) to investigate and quantify the spatial and temporal dynamics of the clogging of the gravel bed at large spatial scales (i.e., in the range between decimeters to meters). The method has the capability to capture transient and progressive infiltrations and depositions of sediments up to 10,000 fps (frames per second). To the authors’ knowledge, this is the first laboratory tool with this ability that can be utilized in an open laboratory environment. The feasibility and measurement performance of the sensor are demonstrated by comparisons with results of an industry-standard gamma ray computed tomography system and by using various sediment fillings with different infiltrating particle sizes. Different mixture conductivity models are tested to convert raw sensor data to the degree of clogging. This is necessary since the conductivity behavior of water–sediment packings still requires validation, and the arranged geometry is not a standard wire-mesh arrangement but represents a rather complex structure. The validation experiments and results are presented, followed by a discussion on the advantages and drawbacks of the developed GravelSens. An outlook on future studies is also provided, followed by a conclusion.

## 2. Materials and Methods

### 2.1. Wire-Mesh Sensors Working Principle and Data Analysis

**Figure 1:**
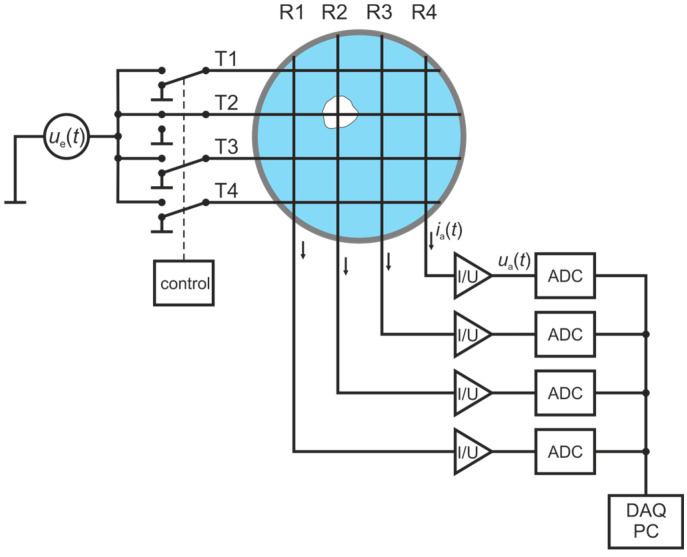
Measuring principle of the wire-mesh sensor (WMS). Four transmitters, T1–T4, are sequentially switched to a bipolar voltage source while four receiver electrodes, R1–R4, measure the current response in parallel, which depends on the local instantaneous dielectric values between the virtual space of the excited transmitters and the receiver electrodes [56].

Wire-mesh sensors (WMSs), first introduced by Prasser et al. [49], are well-established tools to measure two-phase flow phenomena at high spatial and temporal resolution for a wide range of applications, from reactor safety research to oil production and flow assurance up to chemical engineering problems (e.g., flow maldistribution diagnostics in packed beds, optimization of heat transfer processes, monitoring of catalyst distribution) and solar steam generation [51,52]. The technique is based on a matrix-like arrangement of (wire) electrodes. In its standard configuration, a set of parallel wires, acting as a transmitter plane, is stretched over a cross-section of a pipe or a channel, while a second set of parallelly stretched receiver wires, acting as a receiver plane, is mounted with a small axial gap oriented perpendicular to the transmitter plane (Figure 1). By exciting the transmitter wires T1–T4 sequentially, one by one, with a voltage signal and measuring the resulting current towards all receiver wires R1–R4 in parallel, the spatio-temporal distribution of the local instantaneous dielectric properties is obtained as voltage signal $U_{i,j,k}$, where i and j stand for the corresponding receiver and transmitter wires and k for the frame number.

In gas–liquid two-phase flows, where the sensor is predominantly utilized, the local instantaneous phase fractions (e.g., gas or liquid holdup fractions) are derived by taking additional single-phase reference calibration measurements from two different fluids and applying applicable mixture models to the raw WMS data. The simplest mixture model is the so-called parallel model, which assumes a geometry similar to a parallel plate capacitor partially filled with one fluid and partially with another. For a gas–liquid system, the gas holdup fraction is calculated by

(1) $$\alpha_{i,j,k}=\frac{U_{i,j}^{L}-U_{i,j,k}^{\mathrm{meas}}}{U_{i,j}^{L}-U_{i,j}^{G}}$$

with the calibration matrices $U_{i,j}^{L}$ for liquid and $U_{i,j}^{G}$ for gas and the local instantaneous data set $U_{i,j,k}^{\mathrm{meas}}$. Assuming a conductivity-based measurement system, Equation (1) simplifies to

(2) $$\alpha_{i,j,k}=\frac{U_{i,j}^{L}-U_{i,j,k}^{\mathrm{meas}}}{U_{i,j}^{L}}=1-\frac{U_{i,j,k}^{\mathrm{meas}}}{U_{i,j}^{L}}$$

as the measured signal for gas is always zero.

More complex models assume non-linear relations between the measured signal and the calculated liquid holdup. In addition to empirical calculations [57], some well-known models are the serial model, the logarithmic model, and the Maxwell Garnett model. The Maxwell Garnett model is considered more accurate for calculating the holdup of a dispersed phase in a continuous phase (cp. Equation (3)). Although it was originally derived for permittivity measurements, it is also applicable to conductivity-based experiments [58]

(3) $$\alpha_{i,j,k}=\frac{1-U_{i,j,k}^{\mathrm{norm}}}{1+0.5{\cdot U}_{i,j,k}^{\mathrm{norm}}}$$

with the normalized conductance value

(4) $$U_{i,j,k}^{\mathrm{norm}}=\frac{U_{i,j,k}^{\mathrm{meas}}}{U_{i,j}^{L}}$$

For application as a gravel sensor, the WMS technology was utilized here in solid–liquid two-phase flows to quantify the solid (sediment) particle holdup in the bulk water phase. The holdup herein indicates the amount of pore-filling by sediments in the gravel bed and corresponds to the “degree of clogging” within the bed, expressed in percent. Therefore, the assumption that singular particles are suspended in a homogeneous liquid phase seems to be the best fitting. Yet, these models assume a homogeneous mixture within the control volume (region of interest), which is not possible to achieve in reality due to gravity-induced separation. In contrast, a static mixture of water and gravel always comprises a control volume of water, partially filled with densely packed sediment particles with water in between. Therefore, the accuracy of these mixture models for solid–liquid packings needs to be validated experimentally. Thus, an experiment was designed using the developed gravel sensor in a specific mockup channel simulating different static sediment filling scenarios. Gamma ray computed tomography (CT) was employed as a well-established gold standard method for the validation of the new approach.

### 2.2. Gravel Sensor Design and Overview

Initially, a regular grid of seamlessly arranged polyoxymethylene (POM) spheres, each with a diameter of 40 mm (tolerance = ±50.8 µm, density = 1410 kg/m^3^), serves as the basis for the gravel sensor. The sphere size used corresponds to the median particle size (D_50_) values reported in various gravel bed rivers [59,60]. The cubic packing arrangement represents an idealized model of a porous gravel riverbed, providing known and implementable boundary geometries for numerical simulations. This arrangement also provides enough space to implement a vertical stack (h-stack) of three standard WMSs. However, using standard WMS configurations would undoubtedly result in significant blockages of the pores, which necessitated the development of a new approach.

**Figure 2:**
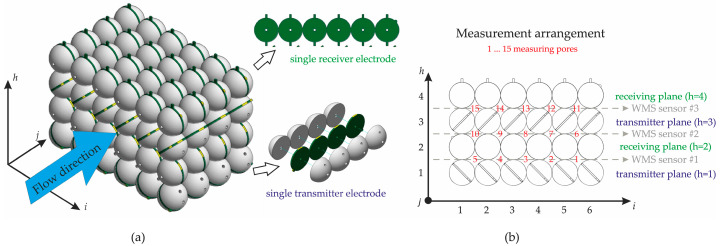
Overview of the gravel sensor (GravelSens): (**a**) 3D visualization; (**b**) measuring area (pore) arrangement of GravelSens that is based on the WMS principle. To ensure non-destructive operation, both the receiver and transmitter wires were realized as side-plated printed circuit boards (PCBs) embedded between the two halves of the spheres.

As shown in Figure 2a, six spheres were arranged in the i-direction, four spheres in the j-direction, and four spheres in the h-direction, resulting in ninety-six spheres in total. The void areas generated within this arrangement are suitable for particle pore-filling measurements and are hereafter referred to as “measuring pores”, representing the sub-channels of the developed gravel sensor (GravelSens). The basic WMS principle was integrated into the sphere grid by embedding the transmitter and receiver electrodes as side-plated printed circuit boards (PCBs) between two sphere halves. As shown in Figure 2b, the transmitter sphere electrodes were integrated with 45°-inclined PCB stripes, each comprising four coherent spheres. In total, two i,j planes, each with six transmitter sphere stripes, were assembled to generate the first and third h planes. Contrary to the transmitter electrodes, the receiver sphere electrodes were integrated into lateral crossing PCB stripes, each comprising eight coherent spheres. Consequently, two i,j planes, each with four receiver sphere stripes, were assembled and stacked between both transmitter planes.

It should be noted that this arrangement of transmitter and receiver electrodes represents three standard WMSs in the h-direction (see Figure 2b). Contrary to its common usage, the flow direction is laterally aligned with the sensor. This configuration results in j = 4 successive measurement planes, each orthogonally arranged in the flow direction, with h = 3 measurement heights and i = 5 lateral positions. Accordingly, a total of 60 measurement areas were configured that can be continuously sampled at a maximum frequency of 10,000 Hz.

It is also important to emphasize that while the conventional WMS belongs to a class of intrusive measurement techniques, the developed GravelSens is inherently non-intrusive due to the integration of sensing electrodes within the spheres simulating the gravel bed.

### 2.3. Gamma Ray Computed Tomography Scanning and Data Processing

For accurate validation of GravelSens, radiation-based computed tomography (CT) is preferred due to its ability to quantitatively and non-invasively deliver a cross-sectional phase fraction distribution. The gamma ray CT scanner, introduced by Hampel et al. in 2007 [61], is specialized for two-phase flow investigations in technical apparatuses, making it an ideal benchmark system for this study. This CT scanner has previously been used to reveal gas phase distributions in liquid phases within various environments, including bubble columns, axial pumps, couplings, rotating packed beds, and trickle bed reactors [62,63,64,65,66,67]. The main advantage of the radiation-based measurement technique is its non-invasive measuring principle, eliminating the need for additional sensor inlets or optical clear accesses, thus ensuring the flow scenario under investigation is not disturbed.

**Figure 3:**
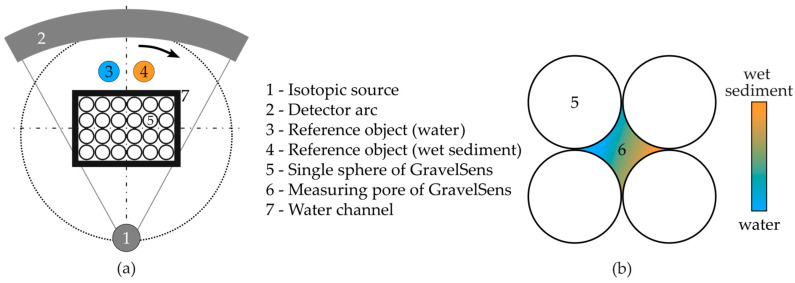
Overview of the gamma ray CT setup: (**a**) principle sketch of CT procedure to non-invasively investigate various sediment classes and sediment fractions; (**b**) a single pore of the gravel sensor (GravelSens) to be investigated.

The CT scanner mainly consists of an isotopic source, ^137^Cs, and a radiation detector that comprises d = 320 single scintillation detectors. Each detector provides an active area of 2 mm in width and 4 mm in height, leading to an in-plane spatial resolution of about 2 mm and an image-segment-averaging height of 4 mm. Both parameters need to be considered concerning subsequent data analysis. To obtain cross-sectional images, radiation projections are acquired from different angular positions relative to GravelSens (Figure 3a). Therefore, the source detector arrangement is continuously rotated around GravelSens by 360°, acquiring a total of p = 1000 equally distributed projections. Accordingly, a radiation intensity matrix from the object $I_{x}(d,p)$ and without object $I_{0}(d)$ is obtained, from which the attenuation matrix

(5) $$E_{d,p}=\ln\left(\frac{I_{x}(d,p)}{I_{0}(d)}\right)$$

is calculated and finally used as input for computed tomography reconstruction algorithms to compute cross-sections of the object $\mu_{i,j}^{meas}$.

Usually, two reference CT scans from the object of investigation are obtained with corresponding single phases, liquid and sediment only, i.e., $\mu_{i,j}^{L}$ and $\mu_{i,j}^{S}$, to be able to quantify phase fractions of CT scans

(6) $$\varepsilon_{sediment}\left(i,j\right)=\frac{\mu_{i,j}^{meas}-\mu_{i,j}^{L}}{\mu_{i,j}^{S}-\mu_{i,j}^{L}}$$

performed at the two-phase operation [67], where $\varepsilon_{sediment}\left(i,j\right)$ represents sediment fraction distribution. This well-established procedure presumes that all components except the two-phase flow remain stationary, requiring careful performance of the reference scans before, between, or after the two-phase CT scan. In the case of GravelSens, a reference CT scan with pure water $\mu_{i,j}^{L}$ and with pure sediment $\mu_{i,j}^{S}$ would be required. This is not possible with the proposed validation experiment mentioned above due to two reasons: (1) Radiation-based computed tomography measures density differences, and thus, it is not possible to provide a 100% sediment filling state of the GravelSens sensor. This is because sediments always contain voids with an effective porosity depending on particle size and distribution. These voids are then filled with either gas or water (Figure 3b). As a result, the effective density of sediment packing significantly differs with the particle size and hence cannot be used as a reference directly. (2) Performing reference scans with GravelSens always means a reassembling procedure of the experimental setup due to sediment cleaning. Thus, the position of GravelSens will inevitably change, and therefore, reference CT scans can no longer be used.

For these reasons, the standard referencing procedure was alternatively performed by using two reference objects positioned aside the GravelSens experimental setup: a glass filled with pure water and another glass filled with fully wetted sediments (see Figure 3). From the reconstructed cross-sectional images, the averaged attenuation value of pure water, i.e., 0% particle fraction, and of wetted sediment ${\bar{\mu }}^{S}$, i.e., the fraction of maximal sediment load, can be obtained for each particle size class, respectively. Assuming each sediment load provides its own but constant effective porosity $\Phi_{sediment}$, the attenuation value in the measuring pores of GravelSens (see Figure 3, #6) varies only (see Section 3.2) between both references by adding more or less sediment fractions, leading to different fillings:

(7) $$\varepsilon_{sediment}\left(i,j\right)=\frac{\mu_{i,j}^{meas}-{\bar{\mu }}^{L}}{{\bar{\mu }}^{S}-{\bar{\mu }}^{L}}$$

Eventually, a weight matrix $w\left(i,j\right)$ is applied to mask only the relevant pixels in the sediment fraction distribution $\varepsilon_{sediment}\left(i,j\right)$ that correspond to the measuring pores in GravelSens. Pixels outside of measuring pores are assigned $w\left(i,j\right)=0$. By summing the weighted values of the pore areas and normalizing these values with the known sediment-specific effective porosity ($\Phi_{sediment}$), the final sediment fraction value

(8) $${\bar{\varepsilon }}_{sediment}=(1-\Phi_{sediment})\cdot \sum_{i,j}w(i,j)\cdot \varepsilon_{sediment}(i,j)$$

is obtained, which is then directly comparable with the measured values of GravelSens.

The effective porosities $\Phi_{sediment}$ of each particle size class were estimated by mixing exactly 1 L of sediment particles and 1 L of water in a calibrated cylinder (laboratory equipment) with a total volume of 2 L. Mixing was achieved by carefully stirring the cylinder manually to eliminate trapped gas bubbles and ensure homogeneous mixtures with the gravel as much as possible.

## 3. Experiments and Results

### 3.1. Gamma CT Setup and GravelSens Accuracy Assessment

**Figure 4:**
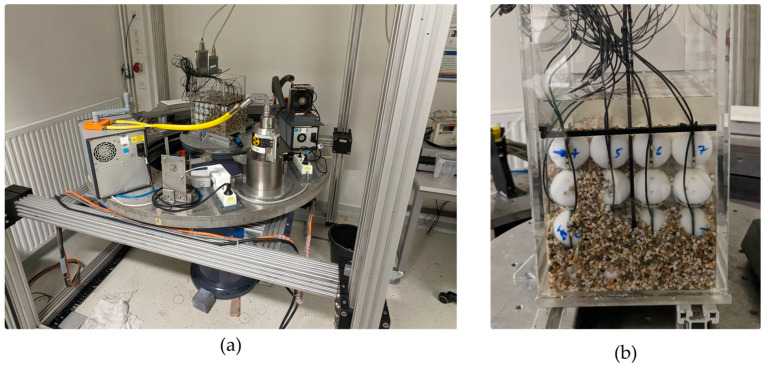
Photographs of the GravelSens calibration experiment: (**a**) the entire setup, including the CT scanner; (**b**) a detailed view of GravelSens partially filled with 3–5 mm sediments.

In order to characterize the accuracy of GravelSens in predicting the degree of clogging as well as to validate the different mixture models from the literature, a static setup was assembled to perform cross-validation with the introduced gamma ray CT scanner. An acrylic channel accommodating GravelSens was placed in the CT scanner (Figure 4).

**Figure 5:**
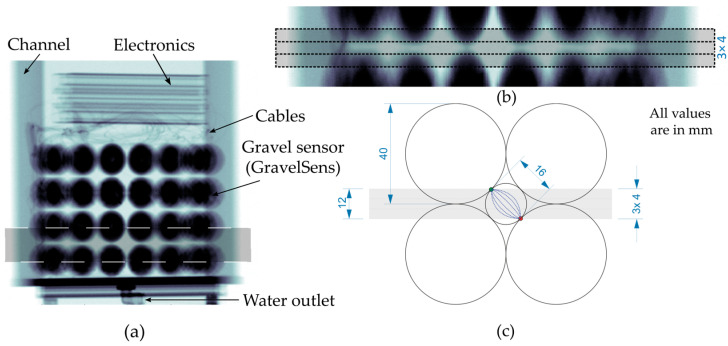
Radiographic scan of the static setup of GravelSens to determine suitable CT scanning planes that cover most of the measuring pore areas at its lowest measuring plane (h = 1): (**a**) entire radiographic scan; (**b**) detailed view of the CT scanning planes; (**c**) a sketch of a single measuring pore with an electrical field depicted.

Initially, a radiographic scan from the static sensor was obtained to identify suitable CT scanning positions. Therefore, the CT scanner was traversed along the height h of GravelSens, and projections p were acquired in steps of 1 mm at a fixed angular position. In Figure 5a, the entire radiographic scan is shown together with a detailed view of the lowest GravelSens measuring plane and the correspondingly selected CT scanning planes (Figure 5b). As the measuring heights of the radiation detector and GravelSens are 4 mm and 12 mm, respectively (Figure 5c), three seamlessly sequenced CT scanning planes were selected to securely cover the entire lowest i,j plane of the GravelSens measuring area. Note that only the lowest GravelSens plane, i.e., at h = 1 (see Figure 2b), was used for validation since it includes in total 20 measuring pores, giving sufficient statistics to check the applicability, i.e., measuring accuracy, of different mixture models.

**Figure 6:**
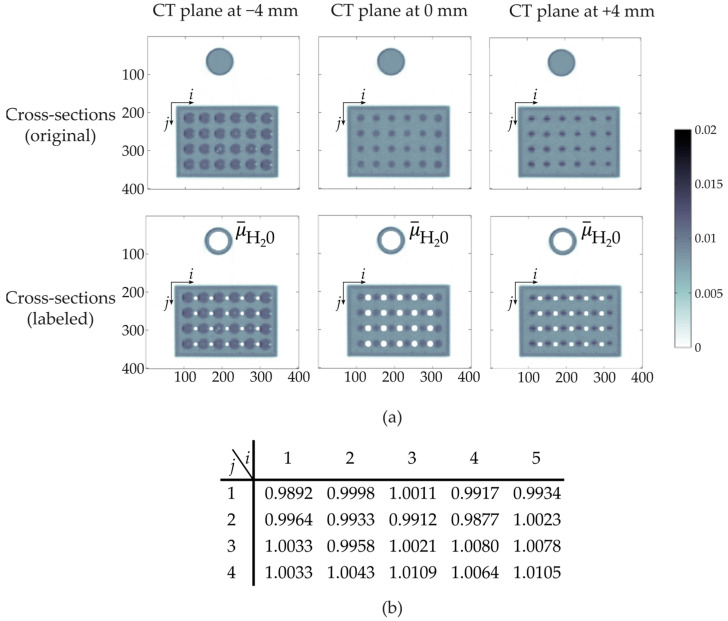
Accuracy of the applied referencing method, as explained in Section 2.3: (**a**) sketch of pixel labeling in all CT scanning planes of the lowest sensor plane (*h* = 1), belonging to each *i*, *j* measuring pore of the completely water-filled GravelSens; (**b**) deviation [%] of the averaged attenuation values of water $\left(\bar{\mu }H_{2}O\right)$ within *i*, *j* measuring pores relative to the averaged water attenuation value of the reference object.

In the next step, the proposed referencing method was quantified. Therefore, CT scans in each defined i,j plane from the GravelSens setup purely filled with water were obtained for 900 s. After image reconstruction, all pixels securely belonging to each measuring pore of GravelSens as well as the reference object were labeled (Figure 6a). Finally, the deviation of the water value for each measuring pore was calculated and determined to be ±1% (Figure 6b).

### 3.2. Validation Experiments

**Figure 7:**
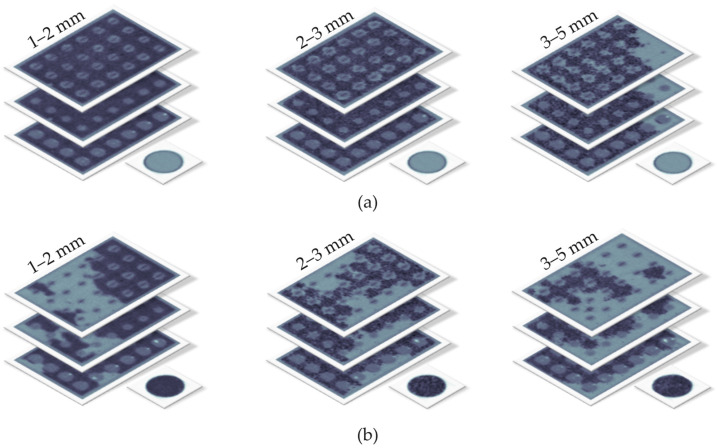
Selected reconstructed CT scans of the lowest measuring plane of GravelSens at different scanning plane heights, particle sizes, and filling levels: (**a**) exemplary scans with dense sediment filling from Experiment 1.1; (**b**) exemplary scans with sparse sediment filling from Experiment 1.2. The gray regions show sediment–water mixture with a dominant water fraction, while the darker areas show the sediment–water mixture with a dominant sediment fraction.

To compare Gamma-CT and GravelSens measurements, the water channel comprising the sensor was filled with three different particle size groups: 1–2 mm, 2–3 mm, and 3–5 mm (Table 1). These particle size groups range from coarse sand to fine gravel, with their calculated effective porosities $\left(\Phi_{sediment}\right)$ being 36%, 38%, and 40%, respectively, based on volumetric measurements. Various filling levels of the measuring pores were achieved by supplying particles arbitrarily, followed by subsequent mixing. This resulted in various clogging configurations, as shown in the reconstructed CT images (Figure 7). Each CT scan took 900 s. Fine sediment clogging measurements were performed with GravelSens before and after the CT scan at a frequency of 5000 fps for a duration of 30 s each. The experiments were repeated for each particle size group (Table 1) in order to cover a wider range of filling conditions. Prior to supplying sediment, GravelSens readings in water were recorded for a duration of 30 s to calibrate the gravel sensor.

### 3.3. Comparisons of Gamma CT and GravelSens Measurements

To validate their particle holdup accuracy and compare the WMS readings, the readings of GravelSens were converted to the degree of clogging using both the parallel model (see Equation (2)) and the Maxwell Garnett model (see Equation (3)). These calculated degree of clogging values in the water matrix, expressed in percent, were then compared with the results from the gamma CT scans according to Equation (8).

**Figure 8:**
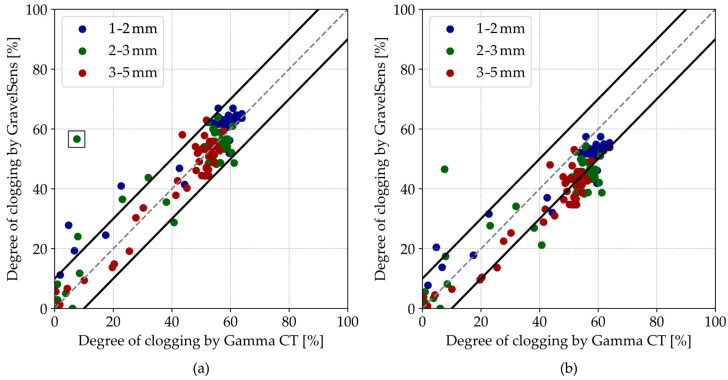
Parity plots of the GravelSens data vs. gamma ray CT data using the (**a**) parallel model and the (**b**) Maxwell Garnett model. The grey dashed lines indicate the 1:1 line, while the black solid lines indicate the ±10% interval.

In general, pore-scale clogging measurements show an overall good agreement between the GravelSens and the CT scans according to the parallel model (Figure 8a). Most data points are within a ±10% range of the 1:1 line of agreement. The theoretically more suitable Maxwell Garnett model slightly and systematically underpredicts the clogging (Figure 8b), which can be attributed to the non-homogeneous particle distribution within the pores. For both models, some deviations from the parity plot are observed, particularly for 2–3 mm sediments. The overestimation by GravelSens can potentially result from direct blockages of the electrodes by individual sediment particles. While the CT technique averages measurements over the entire 12 mm pore space, GravelSens exhibits higher sensitivity close to the transmitters and the receivers. Since the electrical field distribution is inhomogeneous in the sampling area, the measurements can be biased towards the blockage. The correlation for the parallel model is R = 0.92 for the pooled data and R = 0.95 for the pooled data excluding the single outlier (marked with a black square) obtained from the experiment with 2–3 mm sediments (Figure 8a).

**Figure 9:**
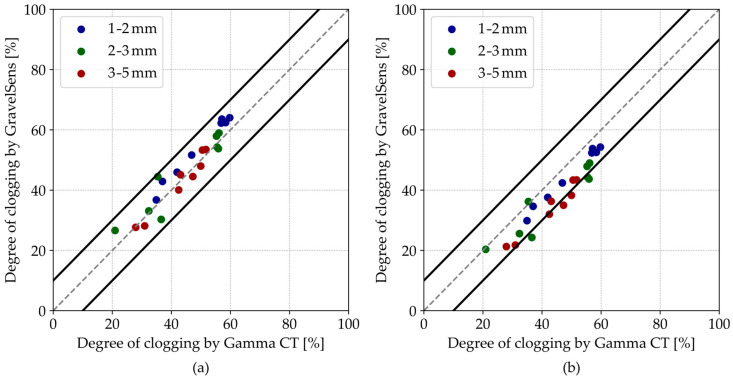
Parity plots of the GravelSens data vs. gamma ray CT data, spatially averaged across the width of the GravelSens, using the (**a**) parallel model and the (**b**) Maxwell Garnett model. The grey dashed lines indicate the 1:1 line, while the black solid lines indicate the ±10% interval.

For further comparisons, the degree of clogging data was also averaged across the width dimension (*i*-dimension in Figure 2a) of the sensor, corresponding to five measuring pores in a line. These spatially averaged results (Figure 9) remain within the ±10% range and would be comparable to the 2D gamma ray scan with a significantly longer data acquisition time, which was proposed for similar applications [46,48].

## 4. Discussion and Outlook

Overall, the results validate and demonstrate the proof of concept and efficacy of the novel gravel sensor (GravelSens) for measuring pore-scale clogging dynamics, with the possibility for gaining an improved understanding for subsurface–surface interactions in rivers. The pore-scale monitoring and visualization tool represents the first in situ laboratory device capable of providing such dynamic insights without disturbing sediment or using ionizing radiation.

GravelSens offers several advantages over GRA, X-ray micro-CT, and MRI techniques, which usually require special facilities and are associated with prohibitively high costs. Importantly, the WMS technique can be employed in hydraulic flumes or any open laboratory environment without relying on ionizing radiation. It offers sufficient temporal resolution (up to 10,000 fps), useful for noise reduction and visualization of transient and progressive clogging processes. Another advantage of the approach is that the sphere tiles used for the sensor are modular, which eases the cleaning from fine sediments. As a drawback, our current approach is limited to the used sphere sizes and to the cubic packing configuration of the bed. However, the flexible technique can potentially be used with other packing configurations and sphere sizes. It should also be noted that even for the cubic packed bed, the detailed processes impacting clogging dynamics are still not fully known. This includes temporal and spatial evolution of clogging in response to different flow and sediment supply characteristics as well as identification of the onset of saturated conditions for the pores, where the particles cannot further deposit [4]. Despite these limitations, it is uncontested that GravelSens can be successfully used to monitor and measure fine sediment clogging simultaneously at multiple interstices of the gravel bed across the cross-sections, allowing for the high-resolution profiling of the spatial and temporal evolution of clogging processes without relying on ionizing radiation or sophisticated expensive devices. The next step would be to demonstrate the performance of the sensor in dynamic open-channel flow conditions with different particle sizes and under different sediment supply scenarios.

While GravelSens was mainly developed for environmental and hydraulic applications of fine sediment clogging in gravel-bed rivers, its practical use extends to a wide range of solid–liquid two-phase flow applications. The ability of GravelSens to monitor fine sediment clogging dynamics makes it also a valuable tool for sediment transport and deposition studies in coastal and marine environments. Similarly, the underlying WMS technology can also be applied to geotechnical investigations, where interactions between soil compaction and groundwater flow are crucial [68]. In food and powder technologies, the measuring technique can be utilized to monitor permeability changes and optimize the manufacturing process [69,70]. Furthermore, while the sensor technology can contribute to the assessment of clogging in drainage systems in construction engineering studies, it may also help in ensuring more efficient operation by detecting sediment or solid accumulation in wastewater filtration systems [71]. Thus, despite its originally intended application, the proposed sensor and its validation in solid–liquid two-phase flow appear to be promising in a variety of environmental and industrial settings, with the potential to improve management and sustainability within these systems.

## 5. Conclusions

We present a smart gravel sensor (GravelSens) that is based on the wire mesh sensor technique, and we demonstrated its capability to non-destructively quantify pore-scale clogging of a cubic packed bed by fine sediments. Despite some limitations, GravelSens represents a significant advance upon existing measurement techniques since it provides high-resolution transient and cross-sectional measurements of clogging within the pores of gravel beds. Therefore, the method has the potential to obtain thorough insights into ongoing clogging processes in gravel-bed rivers, including detailed investigations of the two infiltration modes: unimpeded static percolation and bridging. Given the importance of fine sediment clogging in understanding and modelling flow–sediment interactions and their impacts on riverine water quality and ecological health, as well as in making informed sediment management decisions, the developed method is a valuable addition to hydraulics and environmental geomorphology communities.

## Figures and Tables

**Table 1:** An overview of the validation experiments conducted using GravelSens and CT.

Experiment	Sediment Size [mm]	Filling [%] *	Measurement Points [n]	Effective Porosity [-]
1.1	1–2	53.8–61.8	20	0.36
	2–3	52.3–60.7	20	0.38
	3–5	1.7–53.8	20	0.40
1.2	1–2	0.0–63.9	20	0.36
	2–3	0.0–61.2	20	0.38
	3–5	0.4–57.7	20	0.40

* Filling range calculated from Gamma CT measurements.

## Data Availability

The data can be reached at https://doi.org/10.14278/rodare.3089.
